# Biographical

**Published:** 1886-11

**Authors:** 


### Biographical.

Died, in Dresden, October 11, 1886, Dr. Frank Peabody Abbott, of Berlin.

Dr. Abbott descended from an old and honored New England family. About the year 1840, in the early part of his professional life, he established himself as a dentist in Berlin, being almost, if not quite, the first American to practice that profession in Germany, and so great was his success that he attained a world-wide reputation; and very justly too, for he was a man of sterling integrity, with both natural and acquired ability, his knowledge and skill in the science and practice of his profession was fully up with the times.

Dr. Abbott, though of rather retiring disposition and manner, exercised an influence in his profession for good, equal, if not superior, to many of much greater pretension.

The profession, not only in Europe but in this country as well, has sustained a great, yes, well nigh an irreparable loss. Those who knew Dr. Abbott can not do otherwise than revere his memory, and bear his name on to those who come after.

